# Morphological and Functional Discrepancies in Endocrine Pancreas After Partial Hepatectomy in Dogs

**DOI:** 10.1155/1991/19038

**Published:** 1991

**Authors:** Tetsuya Hirano, Tadao Manabe, Takayoshi Tobe

**Affiliations:** 1 First Department of Surgery Faculty of Medicine Kyoto University Kyoto Japan; 2 M.D., First Department of Surgery Faculty of Medicine Kyoto University 54 Shogoin-Kawaracho, Sakyoku Kyoto 606 Japan

## Abstract

To clarify the changes in pancreatic hormones and their role in the regeneration of the liver after partial hepatectomy, we measured the portal levels of insulin and pancreatic glucagon and their responses to a glucose load after about 40% hepatectomy in dogs. The changes in the A and B cells of the islets of Langerhans were examined histologically. In the early stages after hepatectomy portal insulin levels decreased significantly, and the response of portal insulin to a glucose load was lower than in the control sham-operated dogs. Both islet size and the number of B cells increased significantly after hepatectomy. Portal pancreatic glucagon levels increased significantly after hepatectomy, and the response of pancreatic glucagon to a glucose load was not suppressed. The number of A cells also increased significantly.

Thus, there were differencies between insulin and pancreatic glucagon in their morphological and functional effects after hepatectomy. Although this difference is not clearly understood, there is a possibility that insulin consumption is accelerated in the remnant liver after hepatectomy. Insulin and pancreatic glucagon appear to play important but different roles in the regenerating liver from the morphological point of view.


*HPB Surgery*, 1991, Vol. 3, pp. 103–116
Reprints available directly from the publisher
Photocopying permitted by license only
© 1991 Harwood Academic Publishers GmbH
Printed in the United Kingdom

### MORPHOLOGICAL AND FUNCTIONAL DISCREPANCIES IN ENDOCRINE PANCREAS AFTER PARTIAL HEPATECTOMY IN DOGS

TETSUYA HIRANO, TADAO MANABE and TAKAYOSHI TOBE

*First Department of Surgery, Faculty of Medicine, Kyoto University, Kyoto, Japan.*

To clarify the changes in pancreatic hormones and their role in the regeneration of the liver after partial hepatectomy, we measured the portal levels of insulin and pancreatic glucagon and their responses to a glucose load after about 40% hepatectomy in dogs. The changes in the A and B cells of the islets of Langerhans were examined histologically. In the early stages after hepatectomy portal insulin levels decreased significantly, and the response of portal insulin to a glucose load was lower than in the control sham-operated dogs. Both islet size and the number of B cells increased significantly after hepatectomy. Portal pancreatic glucagon levels increased significantly after hepatectomy, and the response of pancreatic glucagon to a glucose load was not suppressed. The number of A cells also increased significantly.

Thus, there were differencies between insulin and pancreatic glucagon in their morphological and functional effects after hepatectomy. Although this difference is not clearly understood, there is a possibility that insulin consumption is accelerated in the remnant liver after hepatectomy. Insulin and pancreatic glucagon appear to play important but different roles in the regenerating liver from the morphological point of view.

KEY WORDS: Hepatectomy, insulin, glucagon, A cell, B cell

### INTRODUCTION

The pancreatic hormones insulin and glucagon have been reported to be important for regeneration of the liver[1–3] and to be closely related to liver function after hepatectomy[4–6]. The level of plasma glucagon has also been reported to be high after hepatectomy[7], and this is assumed to be one of the hepatotrophic factors[8]. Furthermore, the uptake of glucagon and insulin by the remnant liver has been observed to be accelerated after partial hepatectomy[9]. On the other hand, the level of plasma insulin has been reported to be low[7] or high[10] after hepatectomy. In addition, some reports have described hyperinsulinemia after hepatectomy when animals were fasted too long or the liver was injured[11,12]. So insulin levels after experimental hepatectomy seem to depend on the condition of the animal or of the liver.

In this study, to clarify the differences between insulin and glucagon and the roles of insulin and of glucagon in the regeneration of the liver, we examined the morphological and functional changes in the endocrine pancreas after partial hepatectomy.

---

Correspondence to: Tadao Manabe, M.D., First Department of Surgery, Faculty of Medicine, Kyoto University, 54 Shogoin-Kawaracho, Sakyoku, Kyoto 606, Japan.

### MATERIALS AND METHODS

Fourteen male beagle dogs weighing 11 to 14 kg were used in this experiment. For 8 dogs a 40% hepatectomy was performed by resecting the left lateral and central lobes, and a catheter was inserted into the portal vein under general anesthesia with intravenous sodium pentobarbital (25 mg/kg) after a 12-hour fast. The operation time was about 45 minutes. Blood loss was slight, and systemic conditions were well maintained. Before, and 1, 2, 3, 4, 5 days and 1, 2 and 3 weeks after the hepatectomy, portal blood flow was drawn for base line insulin, pancreatic glucagon and blood sugar determinations after a 12-hour fast via a catheter inserted into the portal vein with the animal awake. Blood was stabilized by aprotinin-EDTA in ice and stored below -40°C. Insulin levels were measured by the two-antibody radioimmunoassay method[13]. Pancreatic glucagon levels were measured by radioimmunoassay with a specific antibody to pancreatic glucagon[14]. Portal blood sugar levels were measured by the glucose oxidase method[15]. Intra-assay and interassay variations for insulin were 5 ± 2% and 10 ± 3%, respectively, and those for pancreatic glucagon were 6 ± 2% and 9 ± 2%, respectively.

Before, and 1 and 2 weeks after hepatectomy with the dog awake and caused as little pain as possible, an intravenous glucose tolerance test (IVGTT, 0.5 g/kg) was performed after a 12-hour fast. The glucose disappearance rate (K), the insulinogenic index (I.I) at 15 minutes and the value of the integrated increased insulin ($\Sigma\Delta$IRI) were calculated. The response of pancreatic glucagon to a glucose load was evaluated by the IRG ratio: pre-glucose load value/post-glucose load value of pancreatic glucagon after 15 minutes.

Biopsies were taken from the distal portion of the right lobe of the pancreas before and 1 and 2 weeks after hepatectomy under intravenous pentobarbital anesthesia (25 mg/kg) after a 12-hour fast.

The biopsied specimens weighed less than 1 g, and the removal of this amount of pancreatic tissue seemed to have no effect on the total function of the pancreas. The specimens were fixed by immersion for 24 hours in 10% neutral formalin solution. The tissue blocks were dehydrated and embedded in paraffin. Four serial sections (6 $\mu$ thick) were cut in the central portion of the specimen, transverse to the axis of the pancreas, and stained with hematoxylin-eosin. In a randomly chosen slide for each stage, 400 islets in the hepatectomized group, and 300 islets in the sham-operated group were examined microscopically by two blinded observers (200 islets/obeserver for the hepatectomized group and 150 islets/observer for the sham-operated group; 25 islets/slide), and the size of the islets and the number of nuclei in the islets were calculated. In the morphometry of the islets, we used a square ruled grid (Nikon, Japan) in the focal plane of the microscope eye-piece projected onto the islet sections. We calculated the size of the islet assuming the cut sections of the islets to be ellipses. In addition, the number of A and B cells examined microscopically by the peroxidase antiperoxidase (PAP) method[16] with 3,3-diaminobenzidine as the chromogen in a DAKO PAP kit (DAKO Corportion, Santa Barbara, CA). The sections were preincubated with 10% normal swine serum for 30 min. The serum was suctioned off, and the sections were incubated for 1 hour at room temperature with the primary antiserum, which had been produced in rabbits. The second antibody was swine immunoglobulins to rabbit immunoglobulins, and the PAP-complex was a soluble complex of horseradish peroxidase and rabbit-antihorseradish peroxidase. Endogenous peroxidase was blocked with 1–2%

hydrogen peroxidase before exposure to the specific antiserum. The specificity of the immunohistochemical reaction was checked by (1) omission of the primary antiserum, (2) absorption test of incubation in primary antiserum pretreated with excess porcine insulin and glucagon, and (3) control staining of the porcine pancreas. All the specimens were counterstained with Meyer's hematoxylin.

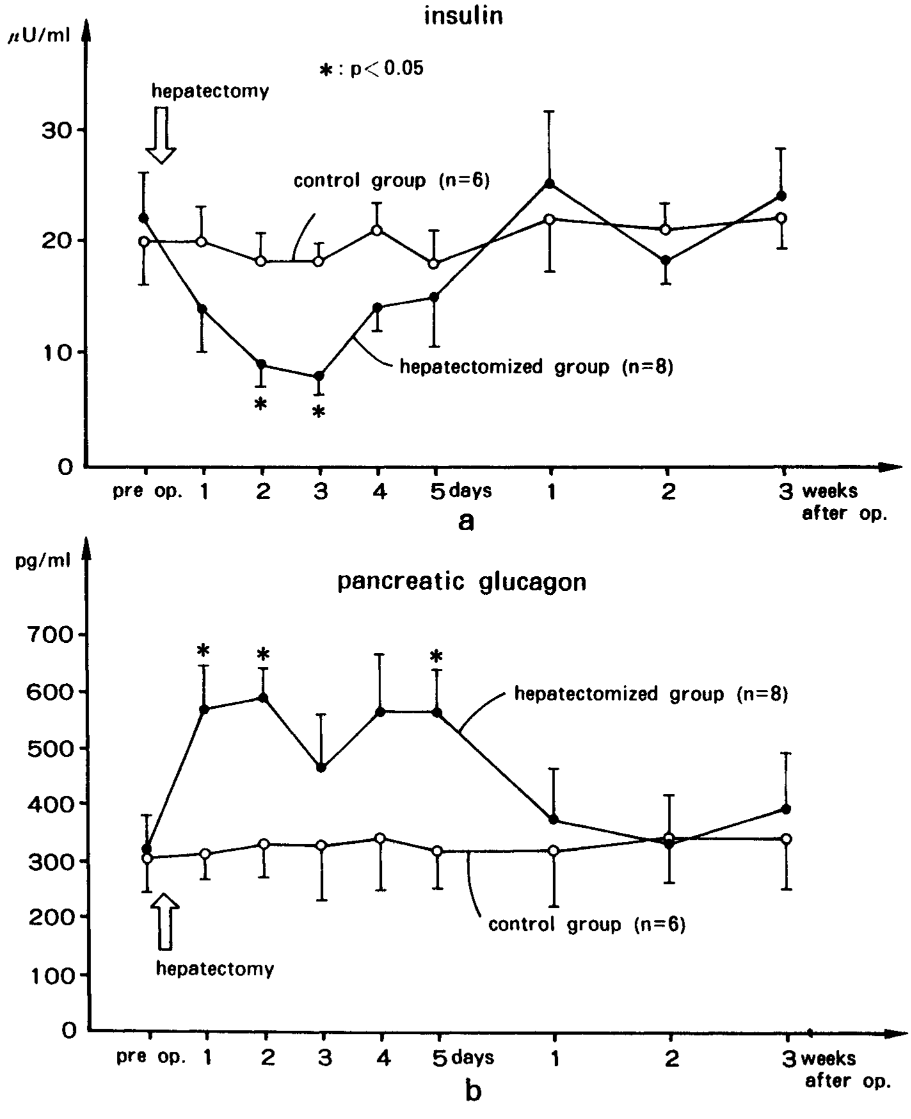

**Figure 1** Changes of (a) portal blood insulin and (b) pancreatic glucagon levels after 40% hepatectomy in dogs. The values are given as the mean ± SEM. The asterisk represents statistically significant differences from the control values.

The control group of six male beagle dogs received sham operations (abdomen opened for 45 minutes, almost as long as in hepatectomy) with the same degree of liver manipulation, but no hepatectomy, and the insertion of a catheter and the same protocol that was used in the hepatectomized group.

During the operation, all the animals were infused with lactated-Ringer solution, 50 ml/h, through a catheter in the right external jugular vein, and this infusion was continued for 6 hours after the surgery. Then all the animals were given free access to tap water and food.

The results of the experiment were evaluated by Student's t-test, and differences of $p<0.05$ were considered significant.

### RESULTS

Figure 1 shows the changes of portal insulin and pancreatic glucagon levels after partial hepatectomy. Portal insulin levels were significantly decreased 2 days ($9\pm2$ $\mu$U/ml, $p<0.05$) and 3 days $8\pm1$ $\mu$U/ml, $p<0.05$) after hepatectomy (controls, $18\pm3$ $\mu$U/ml and $18\pm2$ $\mu$U/ml, respectively). Pancreatic glucagon levels were significantly increased 1 day ($567\pm87$ pg/ml, $p<0.05$), 2 days ($588\pm62$ pg/ml, $p<0.05$), and 5 days ($565\pm83$ pg/ml, $p<0.05$), after hepatectomy (controls, $312\pm42$ pg/ml, $331\pm63$ pg/ml, and $317\pm66$ pg/ml, respectively). Portal blood sugar levels showed no significant changes after hepatectomy (Figure 2).

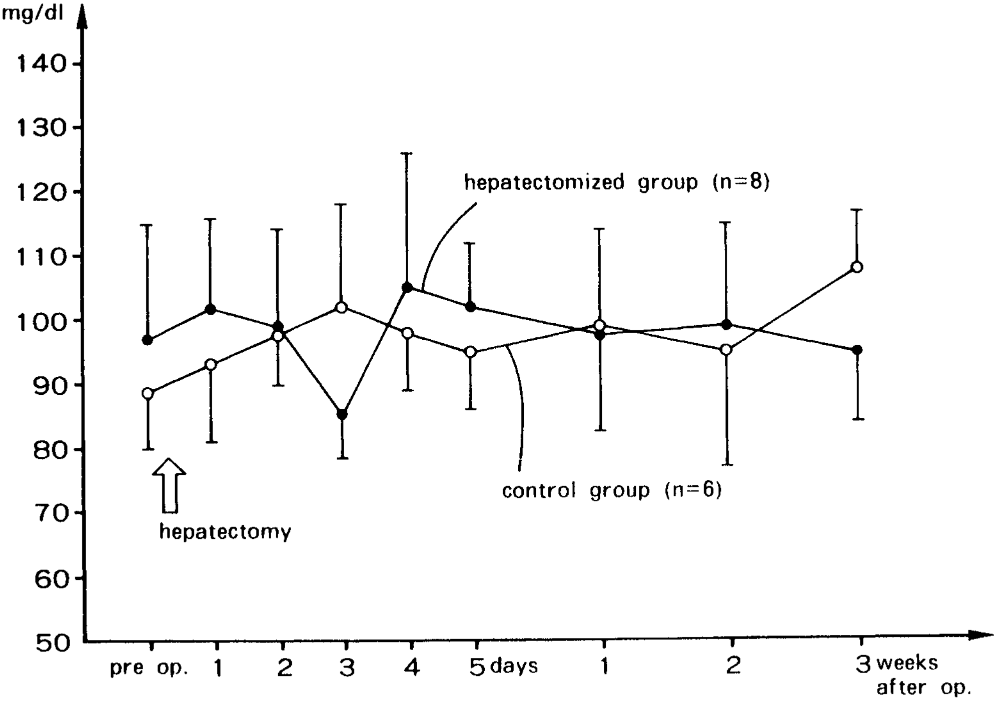

**Figure 2** Changes of portal blood glucose levels after partial hepatectomy in dogs. The values are given as the mean ± SEM. There are no significant differences between the two groups.

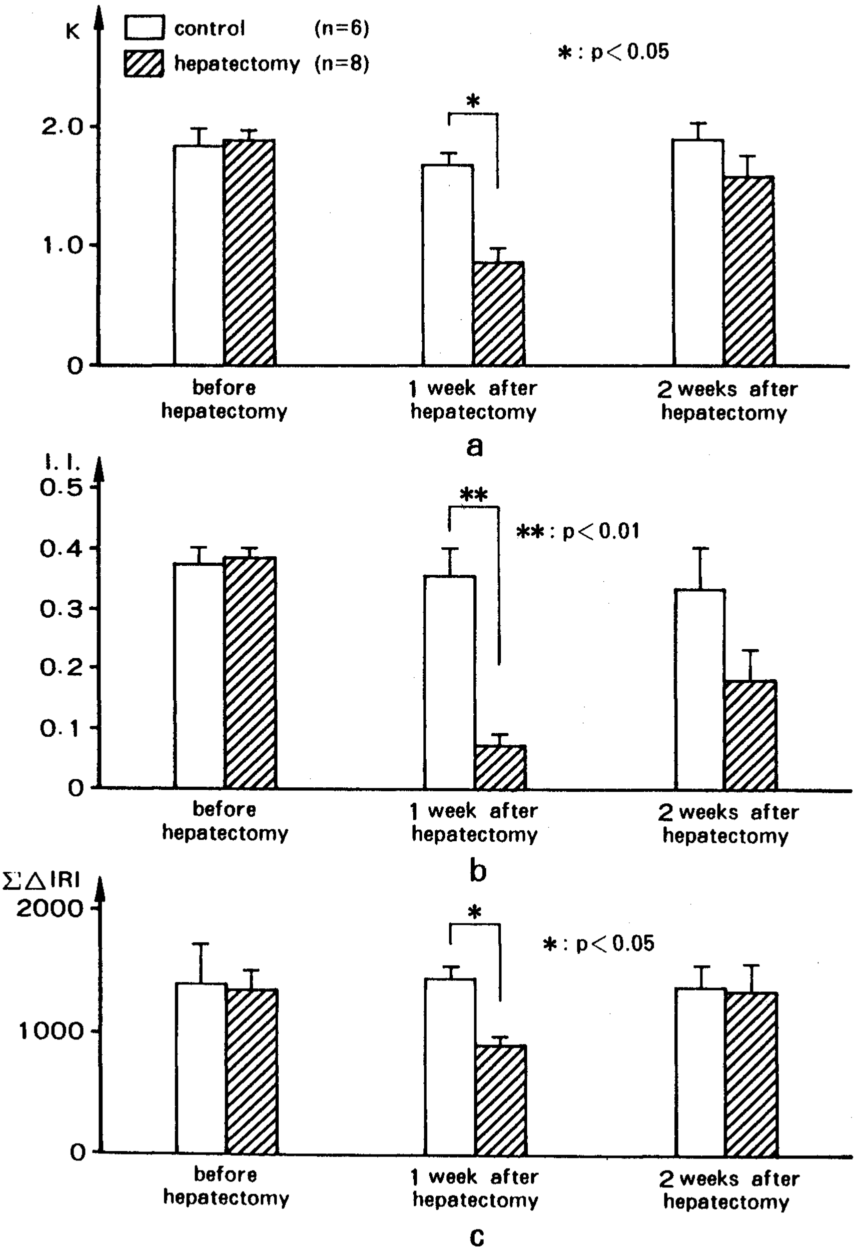

**Figure** 3 Changes of glucose metabolism shown by IVGTT, (a) glucose disappearance rate (K), (b) insulinogenic index (I.I) and (c) integrated increased insulin (ΣΔIRI). The values are given as the mean ± SEM. The asterisks represent statistically significant differences from the control values.

The IVGTT soon after hepatectomy showed a hyperglycemic state and impaired insulin response in the peripheral blood; e.g. a diabetic pattern. The value of K decreased significantly 1 week after hepatectomy ($0.86 \pm 0.12$ vs the control value of $1.67 \pm 0.09$; $p < 0.01$) (Figure 3a). The values of I.I. and ΣΔ IRI also decreased significantly 1 week after hepatectomy (I.I. $0.07 \pm 0.02$, $p < 0.01$; ΣΔ IRI $895 \pm 82$ μU min/ml, $p < 0.05$ vs control group: I.I. $0.35 \pm 0.05$; ΣΔ IRI $1415 \pm 123$ μU min.ml)(Figures 3b and c). Two weeks after hepatectomy, all three of these parameters were almost at their preoperative levels. The IRG ratio was significantly increased 1 week after hepatectomy: $0.97 \pm 0.05$, $p < 0.05$ vs the control group, $0.74 \pm 0.04$ (Figure 4), and the response of pancreatic glucagon to the glucose load was not suppressed.

Morphologically, the size of the islets was significantly increased 1 week ($1214 \pm 35$ $\mu m^2$, $p < 0.001$) and 2 weeks ($2417 \pm 116$ $\mu m^2$, $p < 0.001$) after hepatectomy (control group: 1 week, $726 \pm 33$ $\mu m^2$; 2 weeks, $752 \pm 31$ $\mu m^2$) (Figures 5a and 6). The number of nuclei in each islet was also significantly increased 1 week ($28 \pm 4$/islet, $p < 0.01$) and 2 weeks ($73 \pm 27$ islet, $p < 0.001$) after hepatectomy (control group: 1 week, $22 \pm 3$/islet; 2 weeks, $25 \pm 7$/islet) (Figure 5b). Immunohistochemical studies showed a significantly increased number of A cells 1 week ($13 \pm 3$/islet, $p < 0.001$) and 2 weeks ($21 \pm 7$/islet, $p < 0.001$) after hepatectomy (control group: 1 week, $9 \pm 2$/islet; 2 weeks, $8 \pm 2$/islet) (Figures 7a and b). The number of B cells was also significantly increased at 1 week ($18 \pm 4$/islet, $p < 0.001$) and at 2 weeks ($29 \pm 8$/islet, $p < 0.001$) (control group: 1 week, $13 \pm 2$/islet; 2 weeks, $15 \pm 3$/islet) (Figure 8).

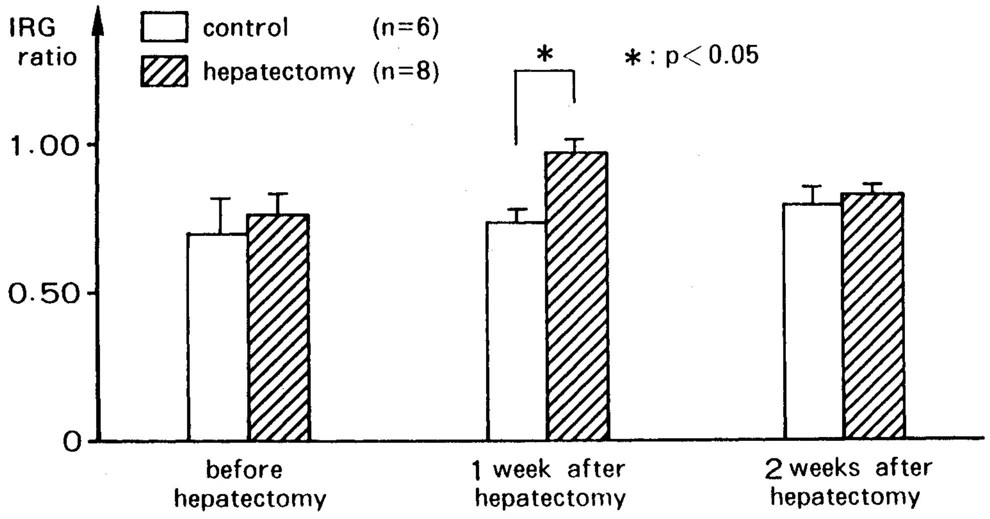

**Figure** 4 Changes of response of pancreatic glucagon to glucose load after partial hepatectomy in dogs. IRG ratio (pre-glucose load value/post-glucose load value) is given as the mean ± SEM. The asterisk represents statistically significant differences from the control values.

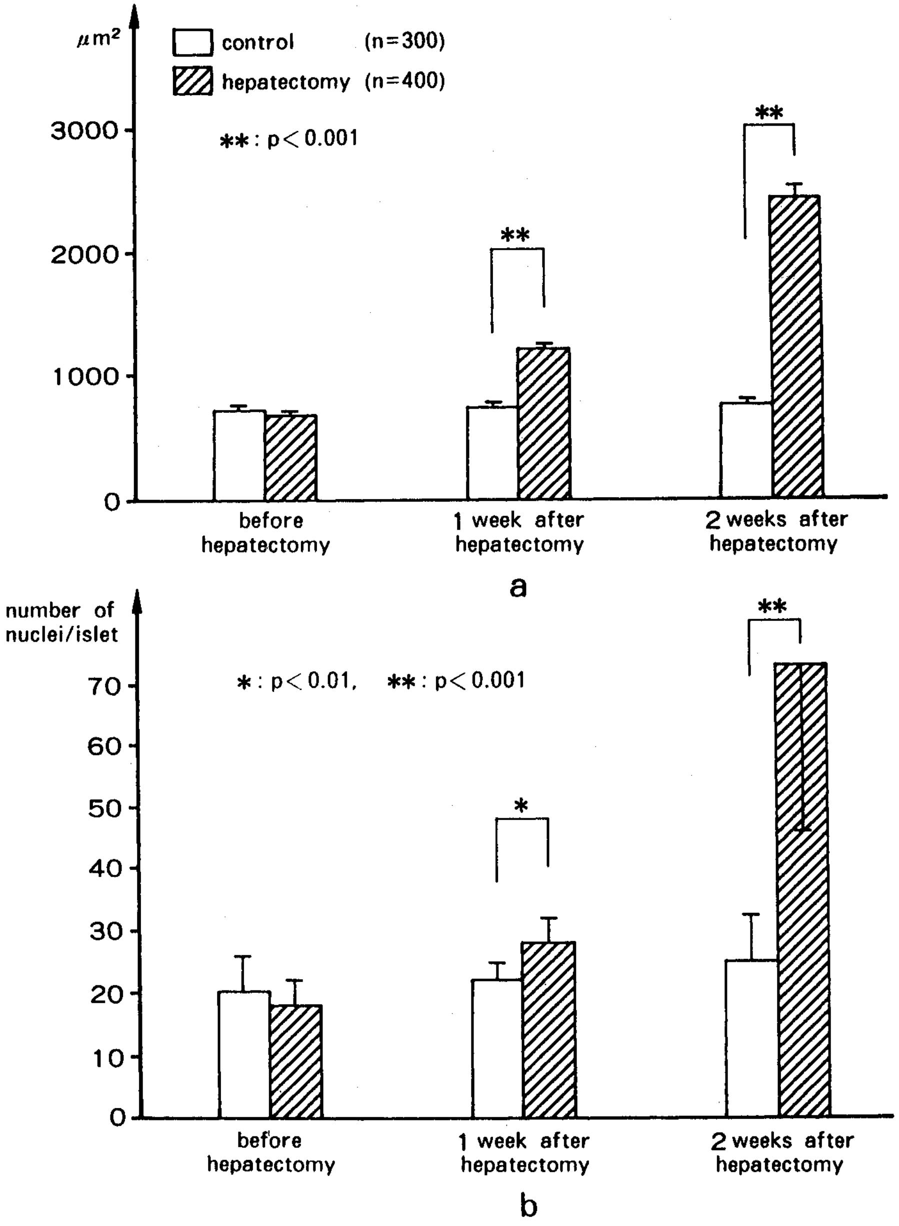

**Figure 5** Changes of islets of Langerhans: (a) the size of the islet and (b) the number of nuclei in the islet are averaged from 400 islets in the hepatectomized and 300 islets in the control groups. The values are given as the mean ± SEM (a) and the mean ± SD (b). The asterisk represents statistically significant differences from the control values.

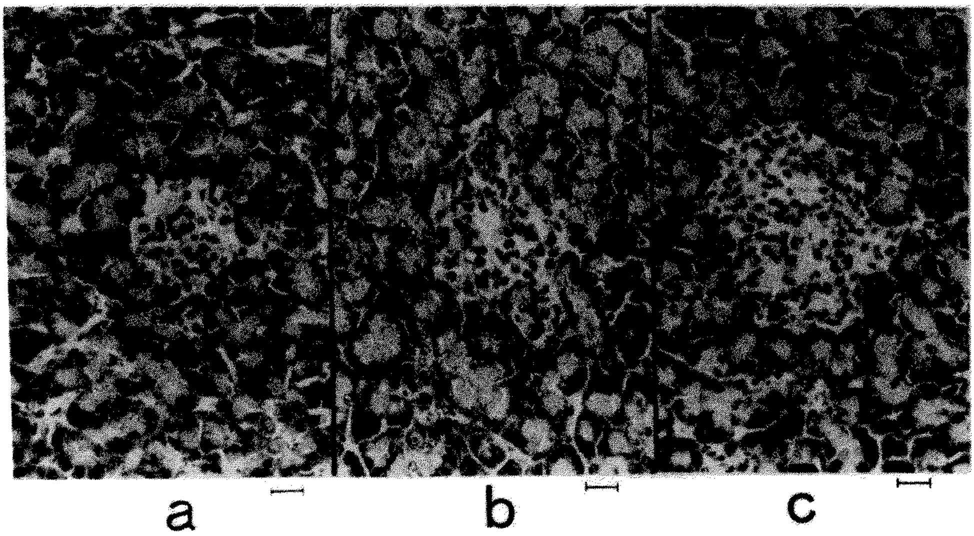

**Figure 6** Microscopic appearance of islets at each stage: (a) before, (b) 1 week after, and (c) 2 weeks after partial hepatectomy (Magnification ×400).

### DISCUSSION

In the early stage after about a 40% hepatectomy, portal insulin levels were lower than in sham-operated controls (laparotomy and liver manipulation). Although we did not measure catecholamine or corticosteroid levels, and we cannot be certain that our sham operation was adequate for the control group, the response of insulin to a glucose load was markedly impaired, as in diabetes, in comparison with the sham-operated control group 1 week after hepatectomy when the insulin levels returned to their baseline in the control group; glucose intolerance continued for about 2 weeks after the hepatectomy, suggesting that the diabetes-like condition persisted longer than ordinary surgical diabetes. Moreover, the islets were enlarged and the number of B cells increased after hepatectomy. This morphological and functional discrepancy suggests that in the early stages after a hepatectomy, the sensitivity of B cells to a glucose load is impaired, or a rather large amount of insulin is consumed in the remnant liver, or some neural effect suppresses the secretion of insulin from B cells, or some of the insulin is consumed in the pancreas itself.

Dogs need a few weeks to recover liver mass and insulin has a primary effect on the membrane transport and intracellular metabolism of glucose[16, 17], insulin also seems to play a central regulatory role in amino acid transport, protein synthesis and control of lipogenesis[18–21] for a few weeks. Accelerated uptake of insulin by the remnant liver also seems to continue for a few weeks even though its level in the portal vein is not high.

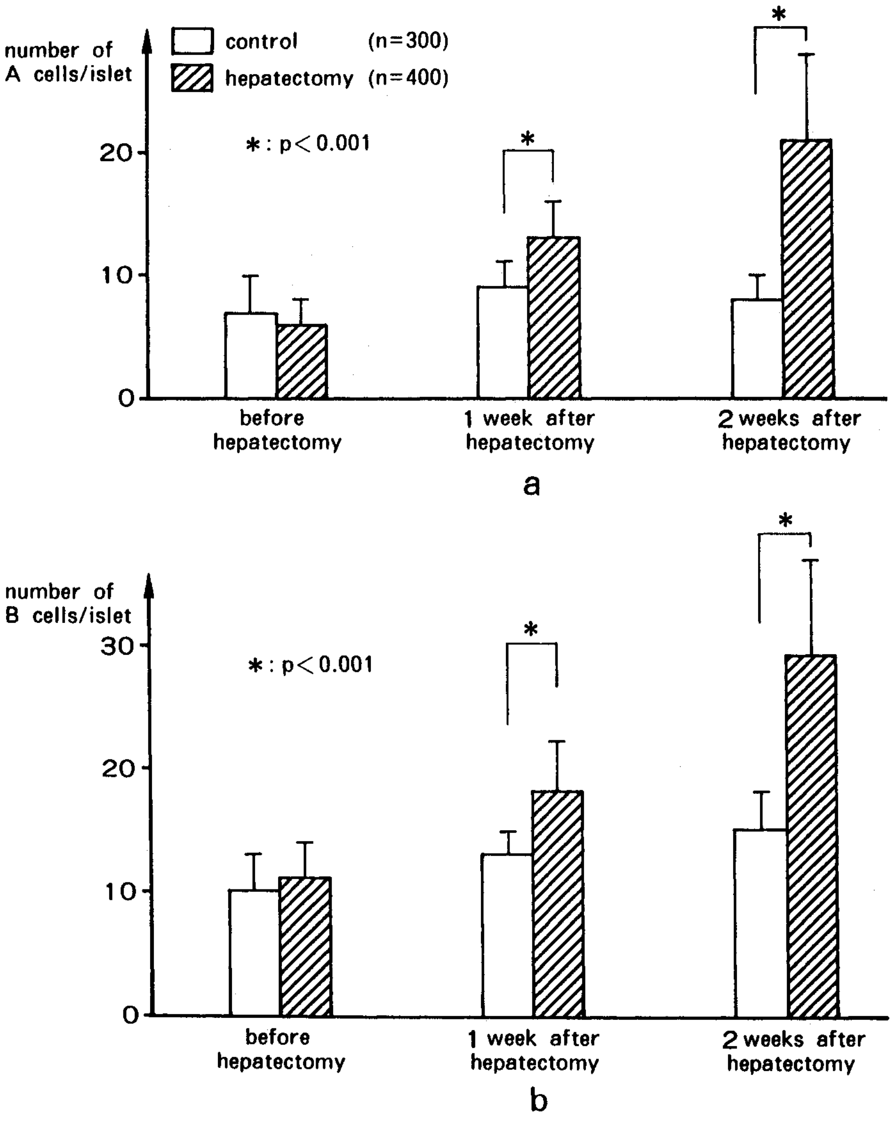

**Figure 7** Number of islet A (a) and B (b) cells averaged from 400 islets in the hepatectomized and 300 islets in the control group. The values are given as the mean ± SD. The asterisk represents statistically significant differences from the control figures.

Moreover, since pancreatic acinar cells have been reported to have receptors for insulin[22,23] and insulin has been said to be important for the activity of acinar cells[24], particularly amylase synthesis[25], it is possible that insulin is consumed in the acinar cells so that these cells can function after hepatectomy. In our recent study in rats, both the amylase concentration in pancreatic tissue and the maxiumum amylase

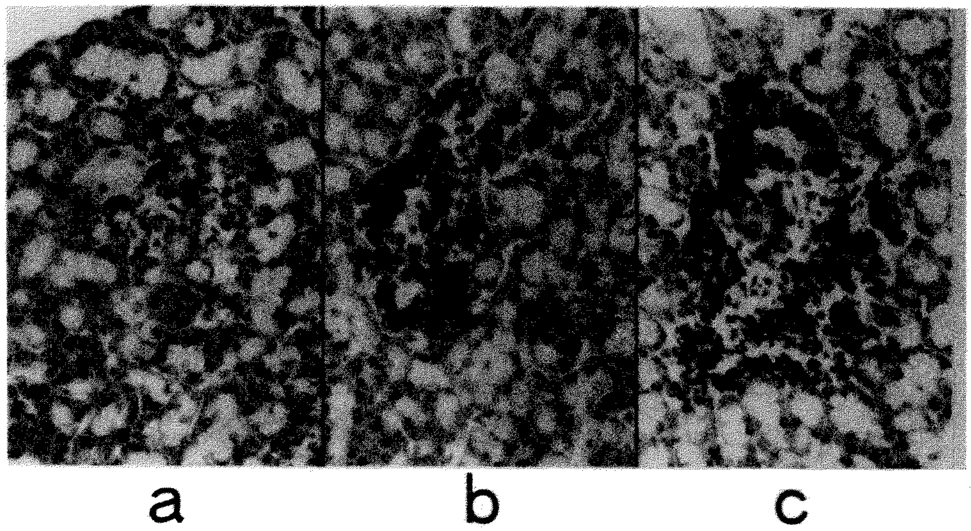

**Figure 8** Immunoperoxidase staining of islet B cells at each stage: (a) before, (b) 1 week after and (c) 2 weeks after partial hepatectomy.

output from dispersed acini increased after hepatectomy[26]. Tenmoku *et al*[27] reported on the intimate relationship between the exocrine pancreas and the liver after hepatectomy, and Rao *et al.*[28] noted increased DNA synthesis both in acinar cells and in islets after hepatectomy. In addition, anatomically, there is a local portal system in the pancreas, the insulo-acinar-portal system[24], and the relationship between the endocrine and the exocrine pancreas has been described as an insulo-acinar axis. Although our present study does not deal with this insulo-acinar relationship directly, insulin seems to play an important role in the pancreas itself as a modifier of the function of acinar cells as well as a hepatotrophic factor to promote the digestion and absorption of nutrients from the gut which are necessary for the recovery of liver mass after hepatectomy.

The reports, cited above and our findings suggest that the impairement of glucose metabolism in the peripheral circulation is due to the consumption of insulin in the pancreas as well as to the accelerated uptake of insulin by the remnant liver.

On the other hand, pancreatic glucagon levels in the portal vein increased, and the response of pancreatic glucagon to a glucose load was not suppressed in the early stages after hepatectomy, and the number of islet A cells were increased. Since the rate of uptake of glucagon by the remnant liver has been reported to be increased after hepatectomy[29], from both the morphological and the functional points of view, it is speculated that pancreatic glucagon is increased and plays an important role in the remnant liver following hepatectomy.

Although both insulin and pancreatic glucagon have been indicated to be hepatotrophic factors, there seem to be morphological and functional differences in their effects. Further studies on the differences between the insulin and pancreatic glucagon levels in the portal vein, vena cava and the remnant liver and direct studies of hepatic[4,5,28] and pancreatic functions with the use of isolated cells and electron microscopy are needed to elucidate this morphological and functional discrepancy in the endocrine pancreas after partial hepatectomy.

*Acknowledgements*

This study was partly supported by a grant, Scientific Research B-62480282, from the Ministry of Education, Science and Culture, and a grant from the Ministry of Health and Welfare of Japan.

*References*

1. Price, J.B.Jr., Takeshige, K., Max, M.H. and Voorhees, A.B.Jr. (1972) Glucagon as the portal factor modifying hepatic regeneration. *Surgery*, **72**, 74–82
2. Bucher, N.L.R. and Swaffield, M.N. (1975) Regulation of hepatic regeneration in rats by synergistic action of insulin and glucagon. *Proceedings of the National Academy Science*, **72**, 1157–1160
3. Starzl, T.E., Francavilla, A., Porter, K.A., Benichou, J. and Jones, A.F. (1978) The effect of splanchnic viscera removal upon canine liver regeneration. *Surgery, Gynecology and Obstetrics*, **147**, 193–207
4. Ozawa, K., Yamada, T. and Honjo, I. (1974) Role of insulin as a portal factor in maintaining the viability of liver. *Annals of Surgery*, **180**, 716–719
5. Johnston, D.G., Johnston, G.A., Alberti, K.G.M.M., Millward-Sadler, G.H., Mitchell, J. and Wright, R. (1986) Hepatic regeneration and metabolism after partial hepatectomy in normal rats: effect of insulin therapy. *European Journal of Clinical Investigation*, **16**, 376–383
6. Johnston, D.G., Johnston, G.A., Alberti, K.G.M.M., Millward-Sadler, G.H., Mitchell, J. and Wright, R. (1986) Hepatic regeneration and metabolism after partial hepatectomy in diabetic rats: effect of insulin therapy. *European Journal of Clinical Investigation*, **16**, 384–390
7. Leffert, H.L., Koch, K.S., Moran, T. and Rubalcava, B. (1979) Hormonal control of rat liver regeneration. *Gastroenterology*, **76**, 1470–1482
8. Bucher, N.L.R. and Wier, G.C. (1976) Insulin, glucagon, liver regeneration, and DNA synthesis. *Metabolism*, **25**, 1423–1425
9. Caruana, J.A. and Gage, A.A. (1980) Increased uptake of insulin and glucagon by the liver as a signal for regeneration. *Surgery, Gynecology and Obstetrics*, **150**, 390–394
10. Cohen, D.M., Jaspan, J.B., Polonsky, K.S., Lever, E.G. and Moosa, A.R. (1984) Pancreatic hormone profiles and metabolism posthepatectomy in dog. *Gastroenterology*, **87**, 679–687
11. Cornell, R.P. (1981) Hyperinsulinemia and hyperglucagonemia in fasted rats during liver regeneration. *American Journal of Physiology*, **240**, E112–118
12. Cornell, R.P. (1981) Endotoxin-induced hyperinsulinemia and hyperglucagonemia after experimental liver injury. *American Journal of Physiology*, **241**, E428–435
13. Morgan, C.R. and Lazarow, A. (1962) Immunoassay of insulin using a two-antibody system. *Proceedings of the Society of Experimental Biology and Medicine*, **110**, 29–32
14. Nishino, T., Kodaira, T., Shin, S., Imagawa, K., Shima, K., Kumahara, Y., Yanaihara, C. and Yanaihara, N. (1981) Glucagon radioimmunoassay with use of antiserum to glucagon C-terminal fragment. *Clinical Chemistry*, **27**, 1690–1697
15. Bruss, M.L. and Black, A.L. (1978) Enzymatic microdetermination of glucagon. *Analytical Biochemistry*, **84**, 309–312
16. Freychet, P. (1976) Interaction of polypeptide hormones with cell membrane specific receptors: studies with insulin and glucagon. *Diabetologia*, **12**, 83–100
17. Godfine, I.D. (1981) Effects of insulin on intracellular function. In: Litwack, G. ed. *Biochemical actions of hormones*. New York: Academic Press 8, 273–305
18. Steiner, D.F. (1966) Insulin and the regulation of hepatic biosynthetic activity. *Vitamine and Hormone*, **24**, 1–61
19. Peavy, D.E., Taylor, J.M., Jefferson, L.S. (1978) Correlation of albumin production rates and albumin mRNA levels in livers of normal, diabetic and insulin-treated diabetic rats. *Proceedings of National Academy Science, USA*, **75**, 5879–5883

20. Yamada, T., Yamamoto, M., Ozawa, K. and Tobe, T. (1977) Insulin requirements for hepatic regeneration following hepatectomy. *Annals of Surgery*, **185**, 35–42
21. Blackshear, P.B. Alberti, K.G.M.M. (1974) Experimental diabetic ketoacidosis. *Biochemical Journal*, **138**, 107–117
22. Korc, M., Sankaran, H., Wang, K.Y., Williams, J.A. and Goldfine, I.D. (1978) Insulin receptors in isolated mouse pancreatic acini. *Biochemical and Biophysical Research Communication*, **84**, 293–299
23. Sjodin, L., Holmberg, K. and Leyden, A. (1984) Insulin receptors on pancreatic acinar cells in guinea pigs. *Endocrinology*, **115**, 1102–1109
24. Williams, J.A. and Goldfine, I.D. (1985) The insulo-pancreatic acinar axis. *Diabetes*, **34**, 980–985
25. Korc, M., Owerbach, D., Quinto, C. and Rutter, W.J. (1981) Pancreatic islet-acinar cell interaction; amylase messenger RNA levels are determined by insulin. *Science*, **213**, 351–353
26. Hirano, T., Manabe, T. and Tobe, T. (1989) Changes in exocrine pancreas after partial hepatectomy in rats. *Medical Science Research*, **17**, 799–800
27. Tenmoku, S., Miyata, M., Fukumoto, T., Ochiai, S., Kasahara, K., Kashii, A., Kanazawa, K. and Iwamoto, Y. (1986) Endocrine and exocrine pancreatic functions following partial pancreatectomy + partial hepatectomy. *Acta Chirurgica Scandinavica*, **152**, 675–679
28. Rao, M.S. and Subbaro, V. (1986) DNA synthesis in exocrine and endocrine pancreas after partial hepatectomy in Syrian golden hamsters. *Experimentia*, **42**, 833–834
29. Watanabe, A., Higashi, T. and Hayashi, S. (1982) Insulin and glucagon in the portal and peripheral blood in liver-injured and partially hepatectomized rats. *Gastroenterologia Japonica*, **17**, 36–41

*(Accepted by S. Bengmark 24 March 1990)*

### INVITED COMMENTARY

Several different approaches have been used to establish that insulin and glucagon might be important hepatotrophic factors after experimental hepatectomy. For example, the two hormones both stimulate DNA synthesis in normal hepatocytes [1] and restore the reduced hepatocyte DNA synthesis after hepatectomy in eviscerated animals[2]. Also, insulin deficiency caused by streptozotocin impairs liver regeneration after partial hepatectomy[3]. Islet hormones thus seem to have the potential ability to be hepatotrophic factors. If, however, the hormones were physiological hepatotrophic factors, augmentation in insulin and glucagon secretion would be expected after hepatectomy. Studies on this topic have demonstrated that, in rats, a standardized 67% hepatectomy induces hypersecretion of insulin in conjunction with exaggerated hepatic uptake of insulin[4]. It could therefore be hypothesized that hepatectomy causes the pancreatic islets to respond with exaggerated insulin secretion; the massively secreted amount of insulin is then largely used already in the liver as a hepatotrophic factor. Similarly in dogs, both limited (42%) and extended (72%) hepatectomy results in hyperinsulinemia, but in this species no obvious change in hepatic extraction of insulin has been observed [5,6]. In contrast, other studies have shown hypoinsulinemia after hepatectomy (see for example 7). When evaluating all the results of different studies in relation to the experimental protocol, a unifying concept has been presented by Cornell: in well-fed animals, the fasting that occurs post-hepatectomy lowers plasma levels of glucose with a concomitant hypoinsulinemia when compared to sham-operated controls, whereas in fasted rats, no hypoglycemia is seen, and then instead the hepatectomy-induced exaggeration of insulin secretion is visible also as an absolute hyperinsulinemia [4]. Thus, the direct effect of hepatectomy on the islets, both in rats [4] and in dogs [5] seems to be induction of hypersecretion of insulin.

Within this research frame, Dr Hirano *et al.* have performed a new study on the effects of limited (40%) hepatectomy on portal levels of insulin and glucagon in dogs. They demonstrate a reduction of plasma insulin levels at days 2 and 3 after the operation, and an elevation of plasma glucagon levels at days 1–5 postoperatively [8]. Their hyperglucagonemia after hepatectomy confirms previous studies [4,5]. However, with regard to plasma insulin levels, their results contrast to those of Cohen *et al.* [5], and their results indicate that hepatectomy does not stimulate but instead reduces insulin secretion. No obvious explanation behind the different results can be found: the two studies both used fasted dogs subjected to 40 or 42% hepatectomy, and though Hirano *et al.* sampled in the portal vein whereas Cohen *et al.* sampled in the saphenous vein, this should not alter the qualitative changes. Very interestingly, Hirano *et al.* also demonstrated a low plasma insulin response to glucose at 1 week after hepatectomy together with a delayed glucose elimination rate, i.e., the partial hepatectomy induced in their model a diabetic pattern.

This strengthens their conclusion that insulin secretion really was impaired after hepatectomy in their model. The impaired insulin secretion was seen when basal plasma insulin levels already had returned to normal values, and this pattern: an initial hypoinsulinemia followed by return to normoinsulinemia despite a persistent impaired glucose-induced insulin secretion shows certain similarities to the characteristics of type 2 diabetes [9]. One difference is, however, that Hirano *et al.* never observed a transient hyperglycemia. In any case, the mechanism behind the hepatectomy-induced diabetes pattern would be of interest to study in more detail. The authors suggest exaggeration of insulin uptake in the exocrine pancreas, due to increased activity in the so called insulin-pancreatic acinar axis [10], a very speculative suggestion. The concurrent enlargement of the islets could be regarded as compensatory hypertrophy after the impaired insulin secretion, though such a compensation is not seen in diabetes [11]. However, also the growth of the islets is regulated by factors [12] that might have elevated plasma levels after hepatectomy.

In conclusion, Hirano *et al.* have presented new data that challenge the concept that hepatectomy is followed by exaggerated insulin secretion of importance for the hepatotrophy of insulin. Future research is now needed along the following lines: 1) how is the detailed regulation of insulin secretion altered at different time period after hepatectomy during the regeneration period? 2) how is the organism protected against hypoglycemia, if insulin hypersecretion evolves for the purpose of hepatotrophy? 3) which mechanisms (growth factors, metabolites, nerves?) govern the islet changes, functionally and morphologically, after hepatectomy? Such studies could give further insight into the understanding of both the regulation of islet function in large and of the metabolic consequences and adaptations to hepatectomy.

Bo Ahrén
Department of Surgery, Lund University, Lund, Sweden

### *References*

1. Short, J., Brown, R.F., Husakova, A., Gilbertson, J.R., Zemel, R. and Lieberman, J. (1971) Induction of deoxyribonucleic acid synthesis in the liver of intact animal. *J. Biol. Chem.* **247**, 1757–1766
2. Bucher, N.L.R. and Swaffield, M.N. (1975) Regulation of hepatic regeneration in rats by synergistic action of insulin and glucagon. *Proc Natl. Acad. Sci. USA* **72**, 1157–1160
3. Johnston, D.G., Johnson, G.A., Alberti, K.G.M.M., Millward-Sadler, G.H., Mitchell, J. and Wright, R. (1986) Hepatic regeneration and metabolism after partial hepatectomy in diabetic rats: effects of insulin therapy. *Eur. J. Clin. Invest.* **16**, 384–390
4. Cornell, P. (1981) Hyperinsulinemia and hyperglucagonemia in fasted rats during liver regeneration. *Am J Physiol* **240**, E112--118
5. Cohen, D.M., Jaspan, J.B., Polonsky, K.S., Lever, E.G. and Moossa, A.R. (1984) Pancreatic hormone profiles and metabolism posthepatectomy in the dog. Evidence for a hepatotrophic role of insulin, glucagon, and pancreatic polypeptide. *Gastroenterology*, **87**, 679–687
6. Itatsu, T., Kishimoto, T., Shintani, T. and Ukai, M. (1979) The release and metabolism of pancreatic hormones after major hepatectomy in the dog. *Endocrinol Jpn*, **26**, 319–324
7. Bucher, N.L.R., Weir, G.C. (1976) Insulin, glucagon, liver regeneration, and DNA synthesis. *Metabolism*, **25**, 1423–1425
8. Hirano, T., Manabe, T. and Tobe, T. (1990) Morphological and functional discrepancies in endocrine pancreas after partial hepatectomy in dogs. *HPB Surgery.*
9. Ward, W.K., Beard, J.C., Halter, J.B., Pfeiffer, M.A. and Porte Jr, D. (1984) Pathophysiology of insulin secretion in non-insulin-dependent diabetes mellitus. *Diabetes Care*, **7**, 491–502
10. Williams, J.A. and Goldfine, I.D. (1985) The insulin-pancreatic acinar axis. *Diabetes*, **34**, 980–986
11. Gepts, W. and In't Veld, P.A. (1987) Islet morphologic changes. *Diabetes/Metabolism Reviews*, **3**, 859–872
12. Portha, B. (1987) Growth pattern of pancreatic B cells. *Front Horm. Res.*, **16**, 102–110

